# Astrocyte-to-neuron intercellular prion transfer is mediated by cell-cell contact

**DOI:** 10.1038/srep20762

**Published:** 2016-02-09

**Authors:** Guiliana Soraya Victoria, Alexander Arkhipenko, Seng Zhu, Sylvie Syan, Chiara Zurzolo

**Affiliations:** 1 Unité Trafic Membranaire et Pathogenèse, Institut Pasteur, 25-28 Rue du Docteur Roux, 75724 Paris CEDEX 15, France

## Abstract

Prion diseases are caused by misfolding of the cellular protein PrP^C^ to an infectious conformer, PrP^Sc^. Intercellular PrP^Sc^ transfer propagates conversion and allows infectivity to move from the periphery to the brain. However, how prions spread between cells of the central nervous system is unclear. Astrocytes are specialized non-neuronal cells within the brain that have a number of functions indispensable for brain homeostasis. Interestingly, they are one of the earliest sites of prion accumulation in the brain. A fundamental question arising from this observation is whether these cells are involved in intercellular prion transfer and thereby disease propagation. Using co-culture systems between primary infected astrocytes and granule neurons or neuronal cell lines, we provide direct evidence that prion-infected astrocytes can disseminate prion to neurons. Though astrocytes are capable of secreting PrP, this is an inefficient method of transferring prion infectivity. Efficient transfer required co-culturing and direct cell contact. Astrocytes form numerous intercellular connections including tunneling nanotubes, containing PrP^Sc^, often colocalized with endolysosomal vesicles, which may constitute the major mechanism of transfer. Because of their role in intercellular transfer of prions astrocytes may influence progression of the disease.

The conversion of the cellular prion protein PrP^C^ to a misfolded β-rich conformer called PrP^Sc^ underlies a group of neurodegenerative diseases known as transmissible spongiform encephalopathies (TSEs). PrP^Sc^ is self-propagating, i.e, capable of inducing the conversion of naïve PrP^C^ molecules to the misfolded conformation^1^ and the accumulation of sufficient levels of PrP^Sc^ results in the formation of oligomers and higher-order fibrillar aggregates. These aggregates may be responsible for seeding the propagation of PrP^Sc^ misfolding between cells following their transfer from one cell to another. The accretion and deposition of prion aggregates in neuronal plaques in diseased brains^2^ results in inexorable and fatal neurodegeneration; however, how these are related is not clear since PrP^Sc^ formation and prion toxicity have been shown to be distinct from each other^3^,^4^,^5^.

Furthermore, while neuronal damage and death are well documented in prion diseases^6^,^7^, the role of other cell types in the brain such as microglia and astrocytes are less understood. We decided to address the role of astrocytes in intercellular PrP^Sc^ transfer and disease propagation for many reasons. Firstly, astrocytes play a major role in the homeostasis of the brain. Astrocytes can modulate neuronal activity by releasing gliotransmitters and scavenging glutamate, are involved in synaptic support and formation, and physically contact and connect large numbers of neurons^8^,^9^,^10^. More interestingly, astrocytes are migrating cells^11^ and also bridge structures like neurons and vasculature that otherwise cannot communicate^12^, thus inviting the question of whether they could be the key to understanding how prion infectivity crosses the brain-blood barrier. The large numbers of tasks they carry out make them indispensable for normal brain functioning and it is important to understand whether these roles are subverted in the course of neurodegenerative disease and perhaps exploited to transfer infectivity. Interestingly, in neurodegenerative diseases, one well-marked phenotype has been reactive gliosis, including a strong astrocyte response marked by cleavage and upregulation of the astrocyte-specific intermediate filament GFAP. The implications of this reactivity are unclear and may indicate a protective response that in turn could be used to transfer infectivity.

Secondly, there are several indications that astrocytes may be involved in prion propagation. Earlier studies have shown that one of the earliest sites of scrapie accumulation in mice appears to be astrocytes^13^ and immunohistochemistry of infected sheep brains shows the accumulation of scrapie in GFAP-positive structures^14^. Primary cerebellar astrocyte cultures from transgenic mice expressing hamster PrP^C^ also sustained infection^15^ indicating that astrocytes are capable of supporting prion replication and infection. Transgenic mice expressing hamster PrP^C^ only in astrocytes developed prion disease upon challenge with an inoculum of hamster scrapie strain 263K^16^. The infection of transgenic-hamster PrP^C^ -expressing astrocytes also resulted in the damage of adjacent neurons that did not express hamster PrP^17^, though those neurons were not capable of replicating prion. Thus, astrocyte infection clearly is deleterious to the brain. However, the fundamental question of whether astrocytes are capable of transferring prion infectivity has yet to be answered.

In this study we investigate this question. Using primary cultures of astrocytes and cerebellar granular neurons (CGNs), we first characterize the relative susceptibility of neurons and astrocytes to infection and show that astrocytes from wild type mice are intrinsically infectable and interestingly, appear to be more prone than neurons to prion replication and accumulation of aggregated PrP^Sc^. We then investigate whether there is transfer of PrP^Sc^ between neurons and astrocytes by developing different co-culture systems. We determine that cerebellar astrocytes can take up PrP^Sc^ from infected neuronal CAD cells in a cell-contact dependent manner. Furthermore, infected astrocytes can efficiently transfer PrP^Sc^ to primary cerebellar granule neurons. Interestingly we find that while astrocytes secrete PrP into the medium, this did not result in efficient prion transfer to primary neurons, suggesting that transfer in primary cultures relies primarily on cell-cell contact. Finally, our data support a role for tunneling nanotubes (TNTs) in the intercellular prion transfer from astrocytes.

## Results

### Primary cerebellar astrocytes and neurons are infected with 22L prion

**Figure 1:**
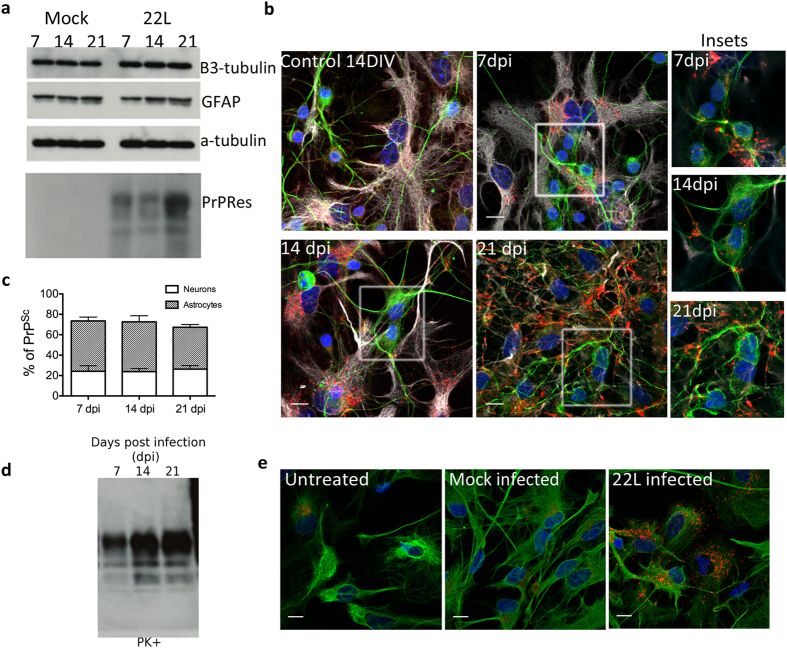
Infection of primary cerebellar mixed cultures. (**a**) Representative western blot of the time course of 22L prion infection. Lowest panel: increase of proteinase-K resistant PrP (PrPRes) at 7, 14 and 21 dpi in the cultures challenged with 22L-brain homogenate (22L) but not those treated with uninfected brain homogenate (Mock). Other panels depict protein levels of other important protein markers within the culture over the time course of infection: the neuronal marker β3-tubulin signal indicates no apparent loss of neurons, astrocyte-specific GFAP levels are constant. Loading control: α–tubulin. (**b**) Representative immunofluorescence (Z-projections) of 22L-infected cultures at 7, 14 and 21 dpi shows that PrP^Sc^ aggregates accumulate over time, mainly in astrocytes. An uninfected CGN culture at 14 DIV shows no aggregation of PrP. DAPI (blue), β3-tubulin (green), PrP^Sc^ (red) and GFAP (white). Insets depict close-ups of neurons at different timepoints of infection. Only the upper z-stacks are taken to reduce the PrP^Sc^ signal from surrounding astrocytes and focus on neuron-associated PrP^Sc^. (**c**) Stacked bar graph comparing the percentage of PrP^Sc^ signal associated with either β3-tubulin or GFAP at 7,14 and 21 dpi. (**d**) Infection of pure cerebellar astrocyte cultures: representative western blot shows accumulation of PrPRes over 21 days of infection. (**e**) Representative immunofluorescence images of astrocytes (marked with GFAP in green) that are either uninfected, mock infected or infected with PrP^Sc^ (red) at 7 dpi. Scale bars: 10 μm.

In order to assess and compare prion replication in neurons and astrocytes, primary mixed cultures of mouse cerebellar granular neurons containing astrocytes were prepared. Since the cerebellum is post-natally developed, cultures often contain around 10–15% of astrocytes at early time-points(7 days *in vitro*, DIV) of the culture; proliferation of astrocytes occurs over time and after 21 days in culture we routinely observe ~30–40%. The mixed cultures were left to differentiate for 5 days before inoculation with 22L prion-infected mouse brain homogenate. Replication of mouse scrapie was monitored by western blot and immunofluorescence at 7, 14 and 21 days post infection (dpi). Western blots (Fig. 1a) revealed a gradual increase of the characteristic proteinase-K resistant PrP (PrPRes) over the time course of the experiment indicating that the CGN cultures were succesfully infected with 22L prion. β3-tubulin signal did not significantly decrease in comparison to the mock-infected cultures (treated with 0.01% brain homogenate from non-infected mice). This suggested there was no major neuronal loss induced by prion infection over this time point. Immunofluorescence studies of these cultures after Guanidium thiocyanate (GdnTCN) treatment to expose PrP^Sc^ epitopes revealed that PrP^Sc^ aggregates could be found in both astrocytes and neurons (Fig. 1b). As we observed no aggregates within astrocytes or neurons 7 days after mock infection (Fig. 1e and data not shown), this suggests that the punctate signal we see is not from aggregated forms of non-infectious PrP^C^, but reveals veritable PrP^Sc^. Interestingly, the majority of the PrP^Sc^ puncta were found within GFAP-positive cells (Fig. 1b,c), suggesting that either astrocytes were taking up the aggregates from neurons (in a possibly protective role) or that they themselves were more apt to replicate prions. Closer inspection of PrP^Sc^ distribution revealed that between 40–50% of the aggregates were within astrocytes compared to approximately 20% in neurons. A large percentage of aggregates (~30%) were unable to be colocalized positively with either type of cell. We hypothesize that this might be extracellular prion aggregate as is frequently reported to occur in infected brain tissue^2^,^17^ although we cannot rule out difficulties in co-labelling.

To confirm that the astrocytes in our cultures do indeed propagate 22L-prion and that the PrP^Sc^ aggregates within them are the result of *de novo* infection following uptake of the infectious seeds, pure cerebellar astrocytes (CA) were isolated and exposed to 22L mouse brain homogenate using the same protocol as for the mixed CGN cultures. Infection was determined as before, by both western blot detection of PrPRes, and immunofluorescence. Figure 1d shows the gradual increase of PrPRes, typical of an infection. Immunofluorescence following guanidium denaturation also showed the canonical punctate distribution of PrP^Sc^ (Fig. 1e), indicating that CA cultures are infected. This is similar to the report by Cronier *et al.* 2004^15^ where pure cerebellar astrocytes over-expressing transgenic hamster PrP were shown capable of sustaining and propagating hamster scrapie infection. The results suggest that mouse scrapie 22L brain homogenate infects both neurons and astrocytes expressing endogenous levels of PrP^C^. They also suggest that cerebellar astrocytes are more susceptible to prion accumulation than cerebellar granule neurons. A very recent report^18^ demonstrated that cortical astrocytes from adult hamster brains were much more efficient than neurons at uptake of exogenous prion, and we speculate that this might promote increased susceptibility of astrocytes to infection.

### Subcellular compartmentalization of PrP^Sc^

**Figure 2:**
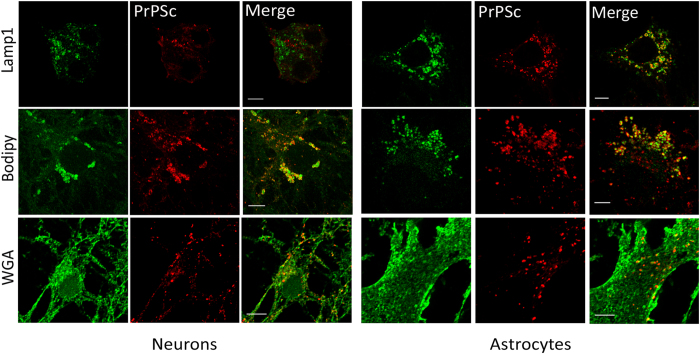
Subcellular distribution of PrP^Sc^ in primary mixed cerebellar cultures. Representative images of colocalization of PrP^Sc^ with different organelle markers in granule neurons (left panels) and cerebellar astrocytes (right panels) from a mixed cerebellar culture at 14 dpi. The images shown for PrP^Sc^ association with the plasma membrane marker WGA are from immunofluorescence on non-permeabilized cultures and hence show external plasma membrane localization. Scale bars: 5 μm.

Studies in immortalized neuronal cell lines^19^,^20^ have shown that PrP^Sc^ is associated with markers of the endo-lysosomal pathway. Furthermore, PrP intracellular trafficking was shown to be important both for prion conversion^19^ as well as in intercellular spreading^21^. Since in our culture system 22L infection affected neurons and astrocytes, we determined the subcellular localization of PrP^Sc^ at the midpoint of infection (14 dpi) in both cell types by using specific neuron and astrocyte markers. In primary mixed cultures, we observed colocalization of PrP^Sc^ with markers of the plasma membrane, lysosomes and lipid droplets (Fig. 2).

In both non-permeabilized and permeabilized cultures, PrP^Sc^ could be found associated with WGA, a common plasma membrane marker, in both astrocytes and neurons (Fig. 2). This suggested that PrP^Sc^ is in close association with the plasma membrane in both cell types. Additionally we noted that this association in neurons occurred quite often along the neurite networks, in string-like patterns that have recently been reported to occur in the neuronal cell line ScGT1^22^. Interestingly, we observed a frequent colocalization of PrP^Sc^ with lysosome markers (Lamp1) in astrocytes, but not in neurons (Fig. 2).

**Figure 3:**
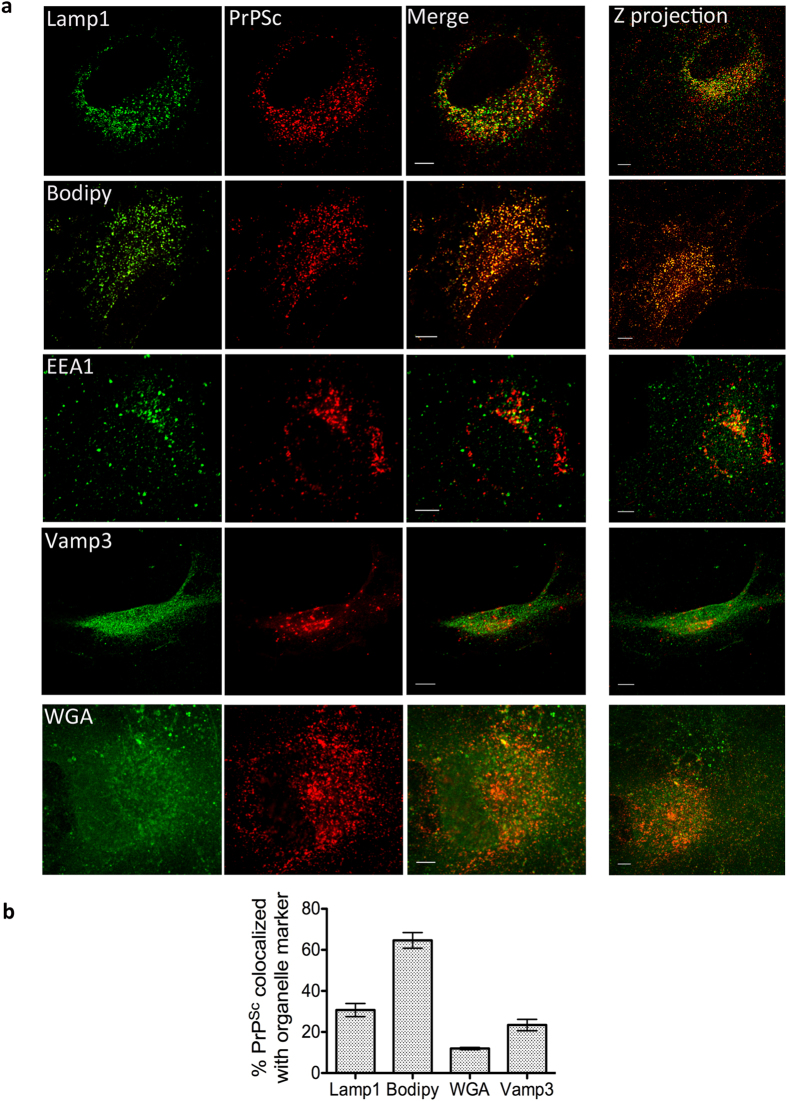
Colocalization in pure astrocyte cultures. (**a**) Representative images of colocalization of PrP^Sc^ with different organelle markers in pure cultures of cerebellar astrocytes at 14 dpi. Single slices are shown for clarity, and the z-projection of the complete cell is shown on the far right. Scale bars: 5 μm (**b**) Quantification of percentage of colocalization of PrP^Sc^ with respective organelle marker in pure cerebellar astrocytes.

These data are consistent with the localization that has been observed *in vivo* in infected murine hippocampi whereby using EM, PrP^Sc^ clusters have been noted on the plasma membrane and in lysosomes of astrocytes in infected neuropil but not in neuronal lysosomes^2^,^17^. Our corroborative results indicate that primary cultures are a physiologically relevant model in which to study prion infections *in vitro*. In addition, we also observed localization of PrP^Sc^ with FL-BODIPY -positive structures in both neurons and astrocytes. As this is a common marker of lipid droplets^23^ this suggests that PrP^Sc^ might associate with cholesterol-rich lipid droplets. We confirmed the subcellular localization of PrP^Sc^ aggregates in pure cerebellar astrocyte cultures wherein we found a large percentage of PrP^Sc^ in lysosomes, consistent with it being degraded, as well as with WGA- and Bodipy-positive structures, similar to the mixed cultures (Fig. 3). PrP^Sc^ was also found in Vamp3-positive compartments. Vamp3 is a marker of the endocytic recycling compartment (ERC), which has previously been shown to be involved in prion conversion^19^. While we could colocalize PrP^Sc^ to EEA1, the percentage of this colocalization was almost negligible (~3%) and therefore considered insignificant.

Of note, we also observed that the lysosomes in pure infected CA cultures appeared to be slightly smaller than those in the mixed cultures. Upon quantification of lysosomal size in astrocytes from mixed versus pure infected cultures using ICY software^24^, we noted that the median lysosome size was significantly increased in mixed cultures (Supplementary Fig. S1). One possible explanation is that astrocytes within the mixed culture phagocytose portions of infected dying neurons or larger external aggregates and the large lysosomes we see might be phagolysosomes resulting from the phagocytic clearance of PrP^Sc^ aggregates.

### PrP^Sc^ aggregates transfer between astrocytes and neuronal cells

The above results show that propagation of PrP^Sc^ could occur in both mixed CGN cultures and astrocyte cultures upon the exogenous application of a PrP^Sc^ source. This mode of infection presumably occurs by endocytosis of the infectious seed by both cell types followed by replication of the misfolded protein inside the cells. We were interested however, in determining if PrP^Sc^ aggregates, once internalized and replicated in one cell type could transfer between astrocytes and neurons and whether this transfer would contribute to the propagation of prions in the culture.

**Figure 4:**
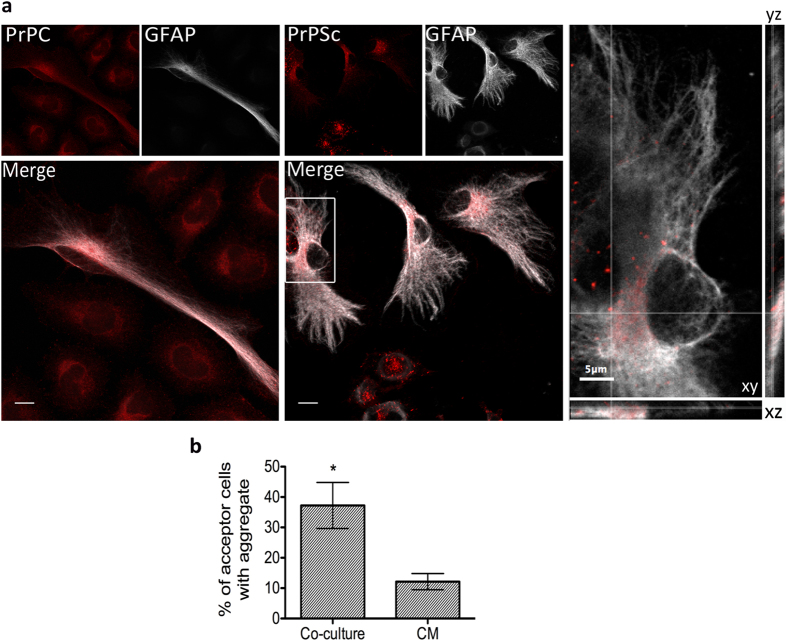
Transfer of prion from infected neuronal cells to astrocytes. (**a**) Left panel: maximum intensity Z-projection image of 24 h co-cultures of uninfected astrocytes and CAD cells showing diffusely distributed PrP^C^. Right panel: immunofluorescence of 24 h co-cultures of astrocytes with prion-infected ScCAD. Larger PrP^Sc^ puncta (red) are clearly visible in infected ScCAD and can be seen to have transferred to the astrocyte on the far left. Scale bars: 10 μm. Inset shows a snapshot of the orthogonal view (*xyz* cuts) through a slice to demonstrate the intracellular localization of PrP^Sc^ aggregates in the astrocyte. (**b**) Quantification of the percentage of acceptor astrocytes with PrP^Sc^ puncta after either 24 h co-culture or treatment with ScCAD-conditioned medium (CM). The results suggest that cell-cell contact is the more efficient method of transfer (*p = 0.0174, Students unpaired t-test).

In order to address whether transfer of PrP^Sc^ could occur from neurons to astrocytes, we set up co-culture experiments between the chronically prion-infected donor neuronal cell line ScCAD and naïve acceptor cerebellar astrocytes. ScCAD was chosen as a “neuron” donor instead of primary cerebellar neurons due to the difficulty in completely eliminating astrocytes from primary CGN cultures, even in the presence of mitotic inhibitors such as FdU. The use of a pure ScCAD culture thus excluded the possibility that any observed transfer to astrocytes resulted from an infected astrocyte within mixed culture donors rather than from neurons. Naïve acceptor cerebellar astrocytes were co-cultured with ScCAD for 24 hours as described in the methods. Immunofluorescence to detect the presence of PrP^Sc^ aggregates in astrocytes revealed aggregates within 37 ± 7.5% of astrocytes (Fig. 4b) within the time frame of our experiment.

In order to confirm that these aggregates derived from the ScCAD donor and did not arise from conversion and aggregation of endogenous PrP^C^ in the acceptor astrocytes from smaller/soluble PrP^Sc^ after uptake, we repeated the same experiment using PrP^−/−^ astrocytes from knockout mice^25^ as acceptors in this co-culture system. We observed PrP^Sc^ aggregates in these astrocytes as well, which strongly support the fact that PrP aggregates transfer from infected CAD cells to astrocytes; they also suggest that PrP^C^ expression in the acceptor cells is not necessary for transfer (see Supplementary Fig S2).

To determine whether transfer was secretion-dependent, we performed experiments wherein conditioned medium from ScCAD cultures were applied on astrocytes for 24 h (see Methods). In this case we obtained 12 ± 2.6% of cells with detectable aggregates (Fig. 4b) suggesting that the transfer of aggregates from ScCAD to astrocytes was much more efficient when there is cell-cell contact.

**Figure 5:**
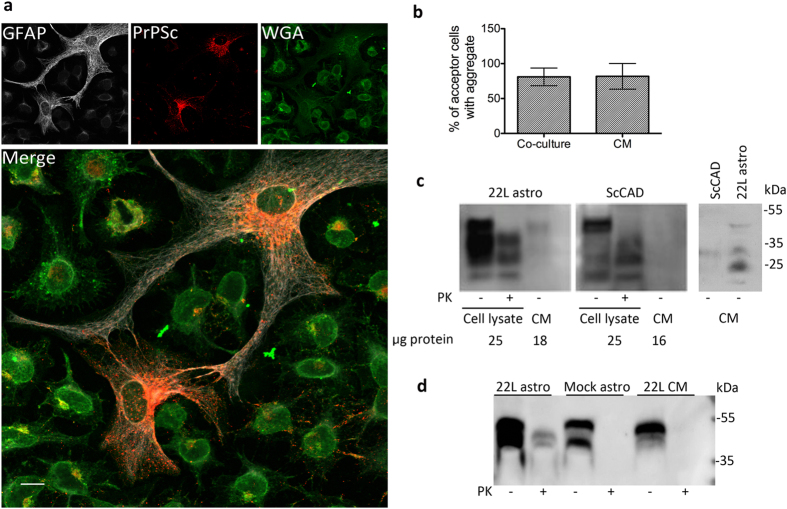
Transfer of prion from infected astrocytes to neuronal cells. (**a**) Maximum-intensity Z projection image of 24 h-co-cultures between 22L-astrocytes and naïve CAD cells. PrP^Sc^ puncta are visible in the majority of CAD cells after co-culture. Scale bar: 10 μm. (**b**) Quantification of percentage transfer after co-culture or after 24 h treatment of CAD with 22L-astrocyte-conditioned medium suggests that transfer from astrocytes to CAD is secretion-mediated. (**c**) Left: immunoblot comparison of the proportion of total PrP (PK-) found in 1/2 the total volume of 24 h conditioned medium (CM) from ScCAD and 22L-astrocyte cultures compared to that found in 25 μg of associated cell lysate. 25μg of lysate corresponds to 1/20th of the total protein lysate of the astrocyte culture or 1/12th of the ScCAD culture. The quantity of protein obtained from methanol precipitation of the indicated volumes of CM is also shown. Far right panel: immunodetection of PrP precipitated from the total volume (3 ml) of CM from confluent flasks of ScCAD and 22L-astrocytes shows very weak signal from ScCAD cultures. (**d**) PrPRes is not detected from 22L-astrocyte CM. Equal quantities of protein (25 μg) from 22L-astrocytes, mock-infected astrocytes and the CM from 22L astrocytes were subjected to Proteinase K treatment.

Next, in order to determine whether astrocytes could transfer PrP^Sc^ to neuronal cells, 22L prion-infected astrocytes (22L-astrocytes) were co-cultured with naïve acceptor CAD cells for 24 h. We consistently observed that a large percentage (81 ± 12.66%, over three independent experiments) of CAD acceptors contained prion aggregates after being co-cultured with 22L-astrocytes (Fig. 5a,b). This was not limited to cells in close contact with astrocytes, but was noted even in cells that were relatively further away from astrocytes, though the numbers of aggregate-positive cells reduced with distance from astrocytes and efficiency of transfer also depended on the confluence level of astrocytes. Additionally, the efficiency of transfer was much higher than that observed from neuronal cells to astrocytes. To determine whether astrocytes were releasing PrP^Sc^ into the medium, CAD cells were incubated with conditioned medium from 22L-astrocytes (see Methods). After 24 h, we observed similar levels of aggregate-positive cells (81.67 ± 18.33%, Fig. 5b), suggesting that astrocytes were indeed secreting PrP^Sc^ that was in turn up taken by CAD cells. We therefore precipitated proteins in conditioned medium from cultures of 22L- astrocytes and ScCAD and assayed for the presence of PrP by immunoblot. After 24 h of conditioning we consistently detect PrP in 22L-astrocyte-conditioned medium (Fig. 5c). Since we did not observe any dead cells over this time period in astrocyte cultures, it seems likely that this PrP is either exocytosed or cleaved from the plasma membrane of infected astrocytes, and is not debris resulting from cell death. Using similar experimental parameters with ScCAD cultures, we could not detect PrP in the conditioned medium from most cultures (Fig. 5c) and occasionally they displayed very low PrP signal compared to the astrocytes medium (5c, far right). This suggests that ScCAD releases very low levels of prion into the media or the inconsistent detection by immunoblot is a result of release by dying cells/debris.

Of note, while total PrP is easily observed, proteinase K (PK) resistance assays of astrocyte-conditioned medium did not reveal any detectable amounts of PK-resistant PrP (Fig. 5d). This could imply that the levels of PrP^Sc^ in the medium released from the astrocytes in this time frame were under the detection sensitivity of a Western blot or that secreted PrP^Sc^ is PK-sensitive^26^.

**Figure 6:**
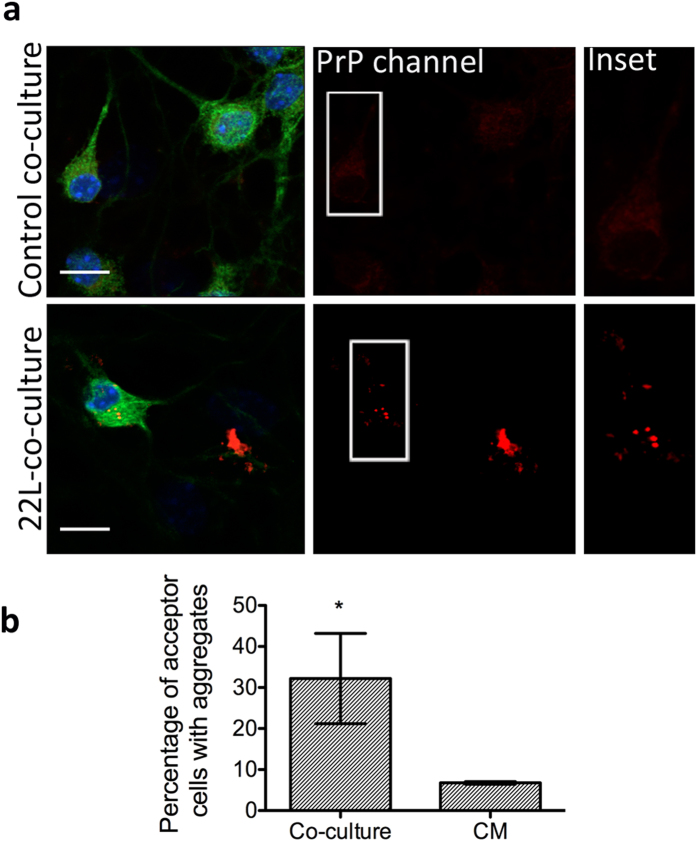
Transfer of prion from infected astrocytes to primary cerebellar granular neurons. (**a**) Upper panels: 11day co-culture between uninfected astrocytes and granule neurons (control co-culture). Lower panels: 22L-astrocytes were co-cultured with cerebellar granular neurons (22L-co-culture) for 11 days. Bright PrP^Sc^ puncta (red) were detectable within β3-tubulin-positive neuronal cell bodies (green) suggesting transfer of infectivity could occur in primary culture as well. Only the upper z-stacks of the image are shown for clarity to reduce signal from astrocytes and focus on neuronal cell bodies. Insets highlight the presence of aggregates within cell bodies of neurons in the 22L co-cultures compared to the more diffuse PrP^C^ signal in uninfected co-cultures. Scale bars: 10 μm. (**b**) Quantification of percentage transfer in co-cultures versus conditioned medium-treated cells. *p = 0.041

In order to support our observation in a more physiologically relevant context, we repeated the co-culture experiments using primary cerebellar granule neurons (CGNs) as acceptors. To this end, astrocytes that had been infected for 7 days were washed thoroughly, enzymatically detached and added to coverslips containing naïve CGNs at 5 DIV. However, after 24 h, it proved difficult to clearly distinguish transferred PrP^Sc^ aggregates from the PrP^C^ signal in the cerebellar granular neurons. A possible explanation is that granule neurons, being very small and with fine neurites, take up the smaller aggregates that are difficult to detect. Thus, to eliminate the possibility of false-positive or false-negative results, we co-cultured the infected astrocytes and naïve neurons for 11 days. Since after 7 days we are able to distinguish PrP^Sc^ aggregates in CGNs after challenging with 22L brain homogenate (Fig. 1), we reasoned that if secreted infectious PrP^Sc^ aggregates were internalized by neurons, some percentage of them would replicate the prion and the resultant aggregates that developed could be more easily detected by microscopy. After 11 days co-culturing, we were able to observe the occurrence of prion aggregates (Fig. 6) in 32.2 ± 10.98% of neurons suggesting that 22L-infected astrocytes were able to transfer infection to cerebellar neurons. Although remarkable, the efficiency of transfer however was lower compared to that observed in the CAD cells. To test for secretion, we performed parallel experiments wherein we conditioned media from 22L-astrocytes for 11 days to approximate the PrP concentrations that might be released over the co-culture time period, and then added this to naïve CGN cultures and incubated for 11 days. Intriguingly, we observed significantly less PrP-aggregate-positive neurons upon incubation with conditioned medium (6.7 ± 0.32%, p = 0.041). Additionally, astrocytes that are present in the mixed acceptor CGN culture, which are usually more susceptible to infection (see Fig. 1), also displayed a low percentage of transfer (11.3 ± 4.03%). These data indicate that while astrocytes release PrP into the medium, transfer via secretion in primary cells is quite inefficient and relies more on cell-cell contact.

**Figure 7:**
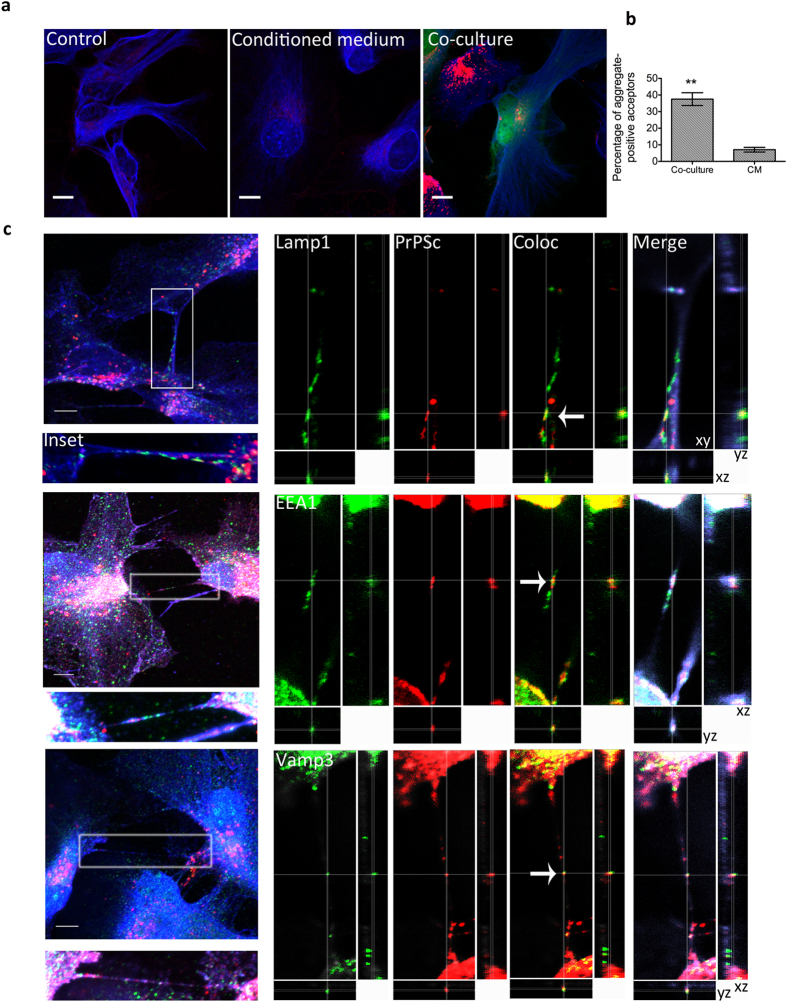
Transfer of prion between infected astrocytes. (**a**) PrP^Sc^ aggregates (red) are easily detected in green CTG-acceptor astrocytes after 24-co-culture with unlabeled 22L-astrocytes (co-culture). Shown for comparison are representative images of uninfected astrocytes (control) and astrocytes that have been cultured for 24 h in the presence of 24 h 22L-astrocyte conditioned medium (conditioned medium). GFAP is shown in blue. Scale bars: 10 μm. (**b**) Quantification of the percentage of astrocytes containing transferred PrP^Sc^ after 24 h co-culturing versus those grown in the presence of conditioned medium. **p = 0.0017 (**c**) Left: Z projections of 22L-infected astrocytes forming numerous PrP^Sc^-containing intercellular connections, including TNTs (Insets). Right: Snapshots of orthogonal views (*xyz* cuts through the image) showing a slice through the TNT: PrP^Sc^ aggregates colocalize with endolysosomal vesicles within TNTs (white arrows), including lysosomes (Lamp1), early endosomes (EEA1) and endocytic recycling compartments (Vamp3). Images for Vamp3 and EEA1 have had their orientation rotated 90° and inverted for presentation purposes. Organelle markers: green, PrP^Sc^: red, and the plasma membrane marker WGA: blue. Scale bars: 5 μm.

Finally, to determine whether astrocytes can also transfer prion between themselves, we co-cultured 22L-astrocytes with wild-type acceptor astrocytes that had been labeled with Cell-Tracker Green (CTG). After 24 h of co-culture, we observed the presence of sharp PrP^Sc^ puncta in the CTG-labelled acceptors (approx. 40%, Fig. 7a,b). Using CTG-labelled PrP^−/−^ astrocytes as acceptors also gave similar results (see Supplementary Fig. S3), thus these appear to be transferred aggregates. We performed the usual conditioned medium controls in parallel and observed very low numbers of aggregate-positive astrocytes that had been exposed to 22L-astrocyte conditioned medium for either 24 h (7.01 ± 1.4%) or 11 days (approx. 13%, data not shown). These numbers were not significantly affected by increasing the time of conditioning from 24 h to 11 days, suggesting that secretion-and-uptake was not very efficient as a mechanism of prion transfer/infectivity in primary cells. We also observed that 22L-astrocytes form numerous intercellular connections in which PrP^Sc^ aggregates can be found. While many of these structures do not strictly fall within the current criteria for tunneling nanotubes (TNTs) (see discussion), nevertheless some proportion of truly TNT-like structures was detectable between astrocytes (Fig. 7c). We found PrP^Sc^ aggregates colocalized with endolysosomal vesicles within TNT-like structures, consistent with what was observed in the case of PrP^Sc^ transfer between neuronal cells^21^. Together with the much higher efficiency of transfer when physical contact between astrocytes was allowed, these data point towards these type of structures to be the predominant method of intercellular PrP^Sc^ transfer.

## Discussion

The intercellular transfer of prion remains a matter of significant interest and interlinks the question of which cell types are involved in spreading prion with that of which molecular mechanisms mediate the transfer. There are multiple routes and different cell types wherein prion is replicated and then transferred from the periphery to the central nervous system^27^. Nevertheless, in all cases (acquired, sporadic or genetic) it is the accumulation and spread of prion in cells of the central nervous system which results in the pathology of disease. However, the role of non-neuronal cells such as astrocytes (which comprise the greater part of the brain) in scrapie infections is unclear, and there are contradictory reports on their effect on neuropathology, depending on the strain of scrapie^3^,^17^. Thus, more detailed research is needed into how cells such as astrocytes influence the course of disease. Additionally, while different mechanisms of prion dissemination have been described and proposed, such as exosomal secretion, tunneling nanotubes, GPI painting or axonal transport^28^,^29^,^30^, it is not yet clear whether these mechanisms are common to all the cell types known to replicate prion or whether different cell types use predominantly one or another form of dissemination depending on their primary functions and physiology. This paper presents direct evidence to suggest that mouse astrocytes are involved in prion intercellular transfer. Our results confirm previous reports that astrocytes replicate prion^15^,^16^, and demonstrates that astrocytes can indeed transfer PrP^Sc^ to neurons as well as between themselves. This is supported by a report demonstrating that prion-infected astrocytes induce neurotoxicity and sensitivity to environmental stress in co-cultured neurons^31^. The authors also observed an overall increase in PrP^Sc^ fluorescence intensity signal upon co-culturing naïve CGNs with ovine scrapie-infected astrocytes for 7 days, suggesting propagation of infectivity. However, the study did not determine the nature of the acceptors (whether neurons or astrocytes) and the extensive neurodegeneration hinders accurate quantification of efficiency of transfer. We observed minimal degeneration in our system, (possibly because in our different co-culture system, neurons are allowed to mature prior to addition of infected donors) allowing direct observation and quantification of intercellular transfer. Additionally, our combined data suggest that while astrocytes can transmit prion infectivity via multiple mechanisms, including secretion, they predominantly use cell-cell contact, such as tunneling nanotubes to mediate transfer. This is likely due to the number of functions astrocytes play: as described in the introduction, they are known to secrete a variety of factors to shape the extracellular matrix and influence neuronal behaviour, however they also rely on direct contact to perform a number of regulatory and protective roles. Thus, these functional roles may be exploited to disseminate infectious PrP^Sc^ to neighbouring neurons.

The colocalization results in the primary mixed cultures show that PrP^Sc^ is differentially localized in astrocytes and neurons with respect to lysosomes. We speculate that this might be related to the functions performed by astrocytes and neurons. Astrocytes may assist certain functions of neurons in order to allow them to focus their cellular energy reserves on their function. For example, astrocytes are responsible for a large percentage of cholesterol production and dissemination in the brain^32^ and neurons are believed to uptake cholesterol from lipoproteins released from astrocytes^33^,^34^. Thus it is possible that astrocytes uptake and degrade protein aggregates from both the extracellular space and from damaged or infected neurons in order to protect them from the deleterious effects of their build-up. Indeed, Chung *et al.*, 2013^35^ demonstrated that astrocytes are capable of phagocytosing parts of neurons. Importantly, cultured astrocytes from different species have been shown to rapidly internalize prion and be capable of its degradation^18^,^36^ and a recent report showed that glia in Drosophila brains could phagocytose Huntingtin aggregates from neurons^37^. Taken together with the observation that astrocytes can take up PrP^Sc^ aggregate from neuronal cells, these data lend weight to the idea that astrocytes may perform most of the prion degradation in mixed cultures, thus explaining the localization of PrP mainly in lysosomes of astrocytes but not neurons.

Nonetheless it is known that neurodegenerative disease impairs astrocytic functions: in mouse models of Alzheimer’s and prion disease, the establishment of disease results in lowered astrocytic degradation capacity^38^,^39^. Thus this function could be subverted in infected astrocytes: the initial capacity to degrade is overtaken at later stages by prion production and increased intracellular burden, resulting in abnormal physiology and, possibly, dissemination from the infected astrocyte. Our finding that PrP can be detected in medium conditioned by infected astrocytes is in line with this idea and suggests that astrocytes release prion protein. Exosomal secretion is one possibility; however it is unclear how efficient this is as a general method of transfer - while prion transfer occurs via exosomes in cultured cell models, the authors note that the efficiency of this method is highly strain-dependent^40^. The authors suggest that this may be due to a strain-dependent size-exclusion effect in packing into exosomes, thereby limiting the efficiency and scope of this method. Interestingly, they observed that the infectivity of 22L prion, which we use in this study, was secreted with one of the lowest efficiencies from cultured cells. Our data with primary astrocytes and neurons suggests that it is equally inefficient as a transfer mechanism in primary cells. Additionally, the size of transferred aggregates in acceptor astrocytes in the co-culture system were far too large to be packaged into exosomes and most likely arise not from short-range secretion but from active transfer of PrP^Sc^ packaged in endolysosomal vesicles or, possibly, phagocytosis from infected cell surfaces. However, in apparent contrast to the data in primary cells we observed efficient transfer of PrP^Sc^ from astrocyte-conditioned medium to CAD cells as detected by the presence of PrP^Sc^ aggregates, despite our inability to detect PrPRes in the conditioned medium. One possible explanation for the difference in transfer efficiency between primary cells and CAD could be the relative susceptibility of different cell types to prion infection; cultured CAD cells may endocytose secreted factors more efficiently or are more susceptible to prion conversion/replication than primary cerebellar granule neurons. In the case of primary neurons or astrocytes whose physiology is very different, and wherein cell-cell contact plays a great role in normal functioning, direct physical contact may be the more efficient mechanism of transfer. It is likely that the interplay of signals between the primary neurons and astrocytes influences and encourages their physical contact in a way that is very different from the co-culture system with CAD cells, enhancing transfer via cell-cell contact. Indeed, we found that astrocytes are found in close apposition to neurons, in direct contact with cell bodies. They also form large numbers of intercellular connections between themselves, including TNT-like structures in which we observe PrP^Sc^ aggregates. These thin actin-containing intercellular bridges can connect distant cells of the same type or heterologous cells such as neuronal cells and bone marrow dendritic cells or neurons and astrocytes^29^,^41^,^42^ and mediate the transfer of small molecules, protein aggregates and organelles^29^,^41^,^43^,^44^,^45^ which make them interesting candidates as a general transfer mechanism. We have previously demonstrated that prion travels within TNTs between CAD neuronal cells, as well as between dendritic cells and primary neurons and that this transfer occurs in endolysosomal compartments^21^,^29^,^46^. Our similar finding of prion transfer between astrocytes suggests that TNTs might be a conserved mechanism of intercellular prion transfer. While identifying TNTs between astrocytes and neurons in our CGN system is fraught with difficulty due to the lack of a TNT-specific marker that allows clear identification of this structure from other neuronal processes, the need for cell-cell contact upon transfer from astrocytes to neurons combined with the presence of PrP^Sc^ in TNTs, suggests that this could be one transfer mechanism in this case as well. Studies have demonstrated that immature neurons can form TNTs with astrocytes^42^ thus evidence exists that such a contact is possible between these cell types, at least during development, prior to axonal/dendritic extension. Intriguingly, it was shown in mature differentiated co-cultures that astrocyte-to-neuron Ca2+-transmission occurred through a synapse-independent, physical intercellular contact that had at least some characteristics of a gap junction^47^. Since connexins have been shown to be localized to a subset of TNTs^42^,^48^, this also suggests that TNTs between astrocytes and neurons in developed brains or differentiated primary cultures may exist and be a potent method of intercellular communication. However, in the absence of tools to identify or specifically block TNT formation it is currently difficult to assess the extent to which this mechanism contributes in intercellular prion transfer. Indeed, it is as yet unclear whether the other intercellular connections between astrocytes also represent a different type of TNT or are other structures that mediate transfer and intercellular communication. Further investigation into delineating the exact mechanisms of transfer of prion between astrocytes and neurons should yield useful insights into the propagation of these neurodegenerative diseases as well as in the field of intercellular communication.

## Materials and Methods

### Ethics Statement

C57BL/6J and PrP^−/−^ (*B6;129-Prnp*^*tm1Cwe*^ ) mice were used to obtain primary cultures. All animal experiments and protocols were performed in accordance with regulations set by the Ministère de l’Enseignement Supérieur et de la Recherche, France. These experimental protocols and methods were approved by the institutional committee, Comité d’ethique en éxperimentation animale (CETEA), Institut Pasteur, (project no. HA0025).

### Primary cultures

Cerebellar granular neurons and cerebellar astrocytes (CA) were isolated from 4–6 day-old mouse pups. Cerebella were isolated, meninges removed and washed twice in PBS. After Trypsin-EDTA treatment for 10 minutes at 37°C followed by trypsin inactivation with FBS, 10^5 ^units/ml of DNase I (Sigma Aldrich) were added and the solution triturated with a 5 ml pipette to dissociate tissue. After gentle centrifugation (700 rpm, 7 minutes no brake), supernatant was removed and 5 ml of complete neuronal medium (DMEM-Glutamax, 10% FBS, B27 supplement, N2 supplement, 20 mM KCl and 1% Pen-strep) was added to the pellet. Cells were plated at a density of 150000-cells/12 mm. For cerebellar astrocytes, the procedure was identical. The day after plating, CA cultures were vigorously shaken to remove debris and other types of glia. Plating and maintenance was carried out using DMEM-Glutamax, 10% Horse serum and 1% Pen-strep as the culture medium.

### Cell culture

The mouse catecholaminergic CAD (cath-a-differentiated) neuronal cell line and its chronically scrapie-infected counterpart ScCAD were grown in OPTI-MEM medium supplemented with 10% FBS +1% Penicillin-Streptomycin.

### Infection of primary cultures

22L-infected mouse brain homogenate was sonicated (2 min, 80% amplitude, 5 sec on/2 sec off cycles using a Vibra Cell Bioblock Scientific sonicator) and diluted to a final percentage of 0.01% (v/v) in either neuronal or astrocyte medium before adding to the culture. After 2 days, the medium was either completely replaced with fresh medium in the case of astrocyte cultures or half the volume replaced in the case of CGN cultures. Medium was refreshed every week.

### Proteinase K resistance assays and western blots

To determine infection of primary cultures, cells were lysed in lysis buffer (25 mM Tris pH 7.5, 1% TritonX-100 and 1% β-octyl glucoside). 50μg of protein was treated with 3.75 μg/ml of proteinase K at 37°C for 30 minutes and methanol-precipitated prior to resuspension in SDS-loading dye and running on a 12% Tris-Glycine gel. Western blots were carried out with Sha31 antibody (SPIBio, mouse anti-PrP, 1:5000), β3-tubulin (Sigma Aldrich, mouse 1:5000), a-tubulin (Sigma-aldrich, mouse 1:10000), GFAP (Dako, rabbit 1:5000). Peroxidase-conjugated secondary antibodies to mouse or rabbit were used (GE Healthcare) and blots were revealed with ECL Western Blot detection reagent (Amersham). For assays on conditioned medium (CM), cells were plated in T-25 flasks and grown to confluence. Medium was removed, cells were washed twice with PBS and 3 ml of serum-free media added. After 24 h conditioning, medium was removed and pelleted at 2000 rpm to remove debris. Cells were lysed in 1 ml lysis buffer. 1.5 ml CM was used for detecting PrP in the absence of PK treatment. The other 1.5 ml was precipitated in 3 vols of methanol overnight (−20 °C), pelleted at 6000 rpm for 15 minutes and the protein pellet resuspended in 50 μl of lysis buffer. Protein in lysates and re-solubilised CM were quantified and PK resistance determined as described above.

### Immunofluorescence

primary cultures or co-cultures were fixed with 4% paraformaldehyde. After permeabilisation with 0.1% TX-100, PrP^Sc^ epitopes were revealed by 5 minutes of treatment with 3 M guanidium thiocyanate (GdnTCN) and detected using either Sha31 (Spibio, IgG1, mouse) or ICSM35 (DGen, mouse IgG2bk). Antibodies to different markers were as follows: GFAP to detect astrocytes (Dako, rabbit polyclonal), β3 tubulin to mark neuronal processes (Sigma-Aldrich, mouse IgG2a), Lamp1 for lysosomes (BD Pharmingen, rat clone 1D4B), Vamp3 for endocytic recycling compartment (ERC) (Abcam, rabbit) and EEA1 for early endosomes (a gift from Dr. Marino Zerial, rabbit) were all used at 1:500 dilution in blocking buffer (PBS +10% goat serum). Secondary antibodies were conjugated to Alexa-488 or Alexa-546. BODIPY 490/505 (1:1000 dilution) was used to stain lipid droplets. Alexa488-Wheat Germ agglutinin, WGA (Life Technologies) was used to mark the plasma membrane. Coverslips were sealed with Aquapolymount ™. Images were acquired using a Zeiss LSM700 confocal microscope. A 40X oil objective (NA 1.3) was used to acquire images of infection or transfer and a 60X oil objective for colocalization studies. To discriminate PrP^Sc^ aggregate signal from PrP^C^, after guanidium denaturation to reveal PrP^Sc^ epitopes, we first acquired images of uninfected controls at a detector gain and laser intensity setting to minimize the PrP^C^ signal, then imaged the 22L-infected cultures at exactly the same acquisition parameter settings for the PrP channel. This constitutes an acquisition-level threshold for PrP^Sc^ signal. Post-acquisition image analysis was performed using ICY software^24^. A fluorescence intensity threshold was always applied to the control images to reduce PrP signal intensity to black, then the same intensity cut-off was applied to the corresponding infected images and the fluorescent objects that remain are considered as aggregated PrP^Sc^. These objects can also be detected using the automated Spot Detector plugin on the software, using the same sensitivity thresholds for the control and 22L culture images. Smaller aggregates are detected using a combination of Scale 1 and 2, which detects fluorescent objects between 1–3 pixels. Larger aggregates are detected with the combination of Scale 2 and 3 to detect objects up to 7 pixels. Mock infected or uninfected cultures show no significant aggregate detection (less than 3% detected in both cases). Images are presented as maximum intensity Z projections or as orthogonal views when determination of intracellular localization of an object requires viewing through the x, y, and z-axes of the image.

### Colocalization studies

Colocalization of PrP^Sc^ with different organelle markers was performed at 14 dpi. After fixation and GdnTCN denaturation, immunofluorescence was performed for PrP^Sc^ and different organelle markers. Secondary antibodies to PrP^Sc^ were conjugated to Alexa-546 and secondary antibodies/dyes to the specific organelles were labeled with Alexa-488 or BODIPY 493/503. Z-stack images were acquired using a Zeiss LSM700 confocal microscope with a 63x oil plan apochromat objective (NA 1.4) to eliminate chromatic aberration. Acquisition parameters were close to Nyquist sampling limits, in order to perform image deconvolution. Deconvolution was performed to reduce the point spread function and improve resolution using Huygens Essential software (Scientific Volume Imaging). Colocalization analysis was performed on deconvolved images using the objects-based colocalization plugin in the image analysis software ICY with object sizes set to 3–7 pixels and with an intensity threshold for PrP^C^ signal.

### Co-culture transfer experiments

Transfer experiments were carried out using a simple Infected Donor-to-Acceptor co-culture system that was adapted to use either astrocytes or neuronal cells interchangeably as donors or acceptors. The different combinations of donor and acceptor cell types are described briefly below.

### Neuronal cells-to-astrocytes

To determine if PrP^Sc^ transfer occurred from the chronically infected neuronal cell line ScCAD to naïve astrocytes, cerebellar astrocytes were plated onto poly-D-lysine-coated coverslips and allowed to attach, and differentiate for 5 days. 1 × 10^5^ “donor” ScCAD cells/ml were then added and co-cultured with the astrocytes for 24 h before fixation, GdnTCN treatment to reveal PrP^Sc^ epitopes and immunofluorescence to detect PrP^Sc^.

### Astrocytes-to-neuronal cells

Astrocyte cultures were infected with 22L prion as described above. At 7 dpi they were washed extensively before trypsinising and re-plating on cover slips. After 5 days they were again washed extensively to remove any debris or released PrP^Sc^ before naïve CAD cells were added in Opti-MEM medium at a cell density of 1 × 10^5^/ml. 24 h post co-culture (dpc) cells were fixed and immunofluorescence carried out to detect PrP^Sc^ in acceptor CAD.

### Astrocytes-to-primary neurons

22L-infected donor astrocytes were washed extensively, trypsinised and re-plated on coverslips containing naïve CGNs at 5 DIV. After 11 days of co-culture the cultures were fixed and immunofluorescence performed to detect PrP^Sc^ associated with β3-tubulin-positive structures (neurons).

### Astrocyte-to-astrocyte

Naïve acceptor cerebellar astrocytes were plated on PDL-coated cover slips. After 5 days, they were stained with 15 μM Cell-Tracker Green (CTG-CMDA, from Life Technologies) according to the manufacturers instructions and washed several times with serum-free medium before replacing fresh medium. 22L-infected donor astrocytes were then trypsinised and added and at 24 h, 3d and 11 days after addition, the co-cultures were fixed and immunofluorescence performed. At each time point the number of CTG-labelled astrocytes with detectable aggregates was counted.

Conditioned medium (CM) controls were performed in parallel for all combinations of donor and acceptor. Briefly, 5 × 10^5^ infected donor (22L-astrocytes or ScCAD) cells were plated in 24 well plates and washed extensively to remove any cell debris. 500μl fresh medium/well was replaced and then cells were incubated for 24 h (or 11 days when co-cultures were performed for 11 days). CM from the donors was then collected and pelleted at 2000 rpm to settle any cell debris. The supernatant was then carefully collected and the entire 500μl was added to the acceptors, which were also plated on coverslips in 24 well plates. In the case of primary neuron acceptors, neuronal supplements B27 and N2 were added to the CM prior to addition to prevent cell death. Cells were fixed at the same time points described for the co-cultures and immunofluorescence performed to detect PrP^Sc^.

Images were processed as described above to determine the number of acceptor cells (astrocytes/CAD/neurons) that had detectable aggregates.

### Statistical analyses

All graphs show the mean + s.e.m. from 3 independent experiments (cultures derived from dissections from different litters). Statistical analysis was performed using Prism software. Student’s unpaired t-tests were used to evaluate the significance of all the data. Normal distributions were assumed but not formally tested. All experiments were repeated at least three times and no issues in reproducibility were encountered.

## Additional Information

**How to cite this article**: Victoria, G. S. *et al.* Astrocyte-to-neuron intercellular prion transfer is mediated by cell-cell contact. *Sci. Rep.* **6**, 20762; doi: 10.1038/srep20762 (2016).

## Supplementary Material

Supplementary Information
