# Supplementary material for: Astrocyte-to-neuron intercellular prion transfer is mediated by cell-cell contact
Source: Sci Rep. 2016 Feb 9;6:20762. doi: 10.1038/srep20762 (PMC4746738; doi:10.1038/srep20762)
Supplement: Supplementary Information [file srep20762-s1.pdf]

Supplementary Information for:

Astrocyte-to-neuron intercellular prion transfer is mediated by cell-cell contact.

Guiliana Soraya Victoria, Alexander Arkhipenko, Seng Zhu, Sylvie Syan and Chiara Zurzolo*

Unité Trafic Membranaire et Pathogenèse, Institut Pasteur, 25-28 Rue du Docteur Roux, 75724 Paris CEDEX 15 *Corresponding author: zurzolo@pasteur.fr, Tel: +33 1 40 61 30 62)

Supplementary Figure S1

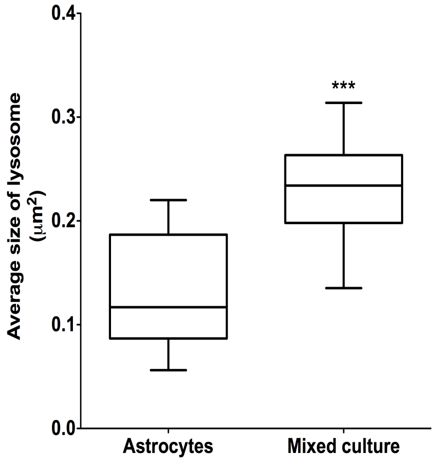

Supplementary Fig S1: Size of lysosomes in astrocytes from 22L-infected pure astrocyte cultures versus in 22L-infected mixed CGN cultures. Lysosomes in mixed infected cultures appear to be bigger than in pure infected primary astrocytes cultures (***$p \leq 0.0001$, unpaired Students two-tailed test). Lysosome size was measured using the aggregates detector plugin on the ICY software.

Supplementary Figure S2

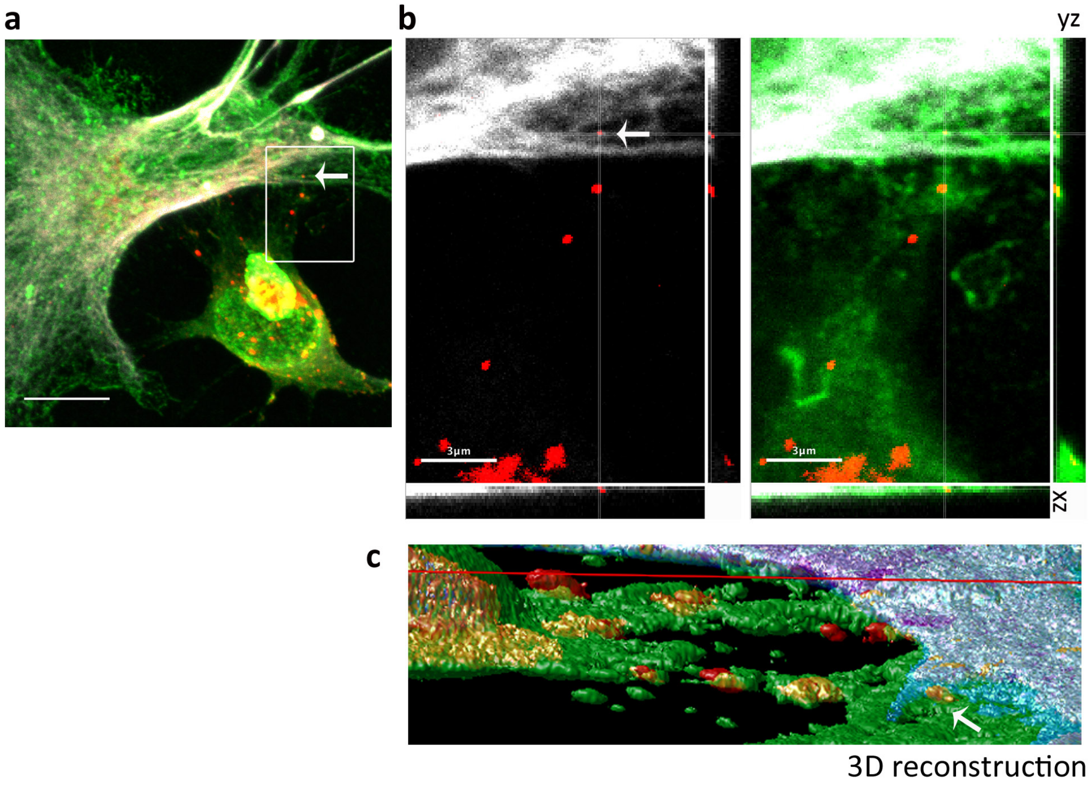

Supplementary Fig S2: Transfer of PrP^{Sc} from chronically infected neuronal cell line ScCAD to $PrP^{-/-}$ astrocytes. **(a)** After 24h co-culture of ScCAD with naïve PrP-deficient acceptor astrocytes, PrP^{Sc} (red) is visible inside astrocytes marked with GFAP (white) Cell boundaries are marked with WGA-Alexa-488 (green). Scale bar : 10µm **(b)** Left panel : orthogonal view of the inset. GFAP (white), PrP^{Sc} (red). The aggregate marked with an arrow clearly localizes within the GFAP-positive astrocyte and is not part of the ScCAD donor cell. Right panel : the same orthogonal view merged with WGA (green) to mark the cell periphery shows that the aggregate is within the astrocyte. **(c)** 3D reconstruction of the image using Huygens Professional software. PrP^{Sc} (red), GFAP (blue-white), PrP^{Sc} (red) and WGA (green). Yellow objects are closely associated PrP^{Sc} and WGA. The arrow highlights the aggregate of interest.

Supplementary Figure S3

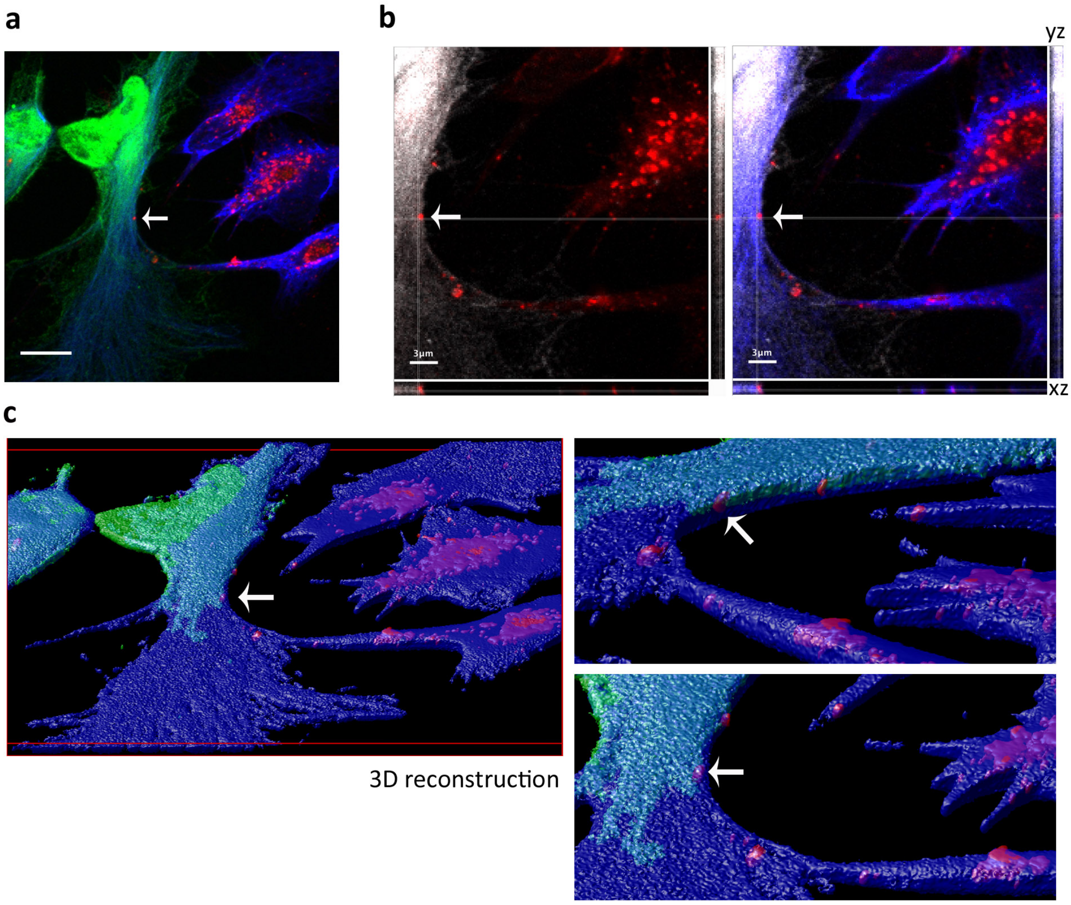

Supplementary Figure S3 : Transfer of PrP^{Sc} from 22L-infected wild type astrocytes to PrP $^{-/-}$ astrocytes. **(a)** 24h co-culture of 22L-infected astrocytes to CTG-labelled acceptor PrP $^{-/-}$ astrocytes (green). Arrow points to PrP^{Sc} aggregate in the CTG-labelled acceptor astrocyte. Scale bars: 10 µm. (**b**) Orthogonal views of the region of interest. Left panel shows CTG (white) to demarcate the acceptor cell and PrP^{Sc} in red. Right panel shows the merge with GFAP (blue) to demarcate the position of the donor astrocytes. **(c)** 3D reconstruction of the image in (a). Different views of the region of interest are shown on the right. Together the images show PrP^{Sc} aggregates visible within TNT/filopodia-like connections arising from the infected donors as well as a transferred aggregate that is localized within the space occupied by cytosolically localized GFAP and green CTG dye in the acceptor (arrow).
